# INVESTIGATING SOCIAL NETWORKS OF OLDER SINGAPOREAN LEARNERS: THE MIXED-METHODS SOCIAL NETWORK APPROACH

**DOI:** 10.1093/geroni/igz038.2768

**Published:** 2019-11-08

**Authors:** Pei-Chun Ko

**Affiliations:** National University of Singapore, Singapore, Singapore

## Abstract

Lifelong learning has been regarded as an important factor of promoting active engagement in later life for researchers and policy makers. Most of the studies tend to illustrate old learners as a homogeneous and self-resilient group of people to engage in lifelong learning. Few studies address older learners’ social capital in affecting their decision of engagement and in sustaining their motivation. The study documented the existing social networks of older Singaporeans in lifelong learning programs and illustrated how social networks contributed their participation in learning. The mixed methods consist of in-depth interviews and two network instruments (Name Generator and Position Generator) based on 30 older Singaporeans (between 50 and 79 years old) who attended lifelong learning courses between 2016 and 2018. Interviews are transcribed and analyzed. The network instruments of are quantified and visualized. The findings show that older learners’ networks included a mixture of social ties from family and friends. Learners’ closeness with network members and their living arrangement with them influenced learners’ involvement in learning and future planning. Single respondents who had more non-kin members in the networks reported to be more active due to their weak ties. Overlapping networks among couple learners increase the spousal support for learning. Learners who had wider ranges of social resources are associated with their interest in learning activities. The study suggests that advocating lifelong learning needs to take older adults’ networks into considerations as networks represent the social forces that influence their decisions and motivations.

